# ZnT2 Is Critical for TLR4-Mediated Cytokine Expression in Colonocytes and Modulates Mucosal Inflammation in Mice

**DOI:** 10.3390/ijms231911467

**Published:** 2022-09-28

**Authors:** Katherine McGourty, Ramya Vijayakumar, Tong Wu, Annie Gagnon, Shannon L. Kelleher

**Affiliations:** Department of Biomedical and Nutritional Sciences, Zuckerberg College of Health Sciences, University of Massachusetts Lowell, 3 Solomont Way, Weed Hall 302, Lowell, MA 01852, USA

**Keywords:** zinc, ZnT2, colitis, colonocyte, TLR4, intestine, inflammation

## Abstract

A wide range of microbial pathogens can enter the gastrointestinal tract, causing mucosal inflammation and infectious colitis and accounting for most cases of acute diarrhea. Severe cases of infectious colitis can persist for weeks, and if untreated, may lead to major complications and death. While the molecular pathogenesis of microbial infections is often well-characterized, host-associated epithelial factors that affect risk and severity of infectious colitis are less well-understood. The current study characterized functions of the zinc (Zn) transporter ZnT2 (*SLC30A2*) in cultured HT29 colonocytes and determined consequences of ZnT2 deletion in mice on the colonic response to enteric infection with *Citrobacter rodentium*. ZnT2 in colonocytes transported Zn into vesicles buffering cytoplasmic Zn pools, which was important for Toll-like receptor 4 (TLR4) expression, activation of pathogen-stimulated NF-κβ translocation and cytokine expression. Additionally, ZnT2 was critical for lysosome biogenesis and bacterial-induced autophagy, both promoting robust host defense and resolution mechanisms in response to enteric pathogens. These findings reveal that ZnT2 is a novel regulator of mucosal inflammation in colonocytes and is critical to the response to infectious colitis, suggesting that manipulating the function of ZnT2 may offer new therapeutic strategies to treat specific intestinal infections.

## 1. Introduction

Colonic infection by bacteria, viruses and parasites results in an inflammatory-type of diarrhea and accounts for the majority of cases presenting with acute diarrhea [1]. Severe cases of infectious colitis can manifest for several weeks and if left untreated may lead to intestinal perforation, megacolon, pancreatitis, renal failure, septic shock and death [1]. Enteropathogenic *Escherichia coli* (EPEC) and enterohemorrhagic *E. coli* (EHEC) are major causes of infectious colitis worldwide [2]. EPEC infection leads to the deaths of hundreds of thousands of children each year in developing countries, while EHEC is a human pathogen responsible for food poisoning and outbreaks of bloody diarrhea and ischemic colitis [3]. EPEC and EHEC are attaching and effacing (A/E) bacteria that adhere to the mucosal epithelium after which a type III secretion system injects virulence factors into the host cell [2]. Following formation of an actin pedestal that supports the bacteria on the mucosal surface, additional effector molecules activate cytoskeletal reorganization, manipulate host cell signaling, and induce apoptosis. While the molecular pathogenesis of these bacterial infections is well characterized, host-associated epithelial factors that affect risk and severity of infectious colitis are less understood.

ZnT2 (*SLC30A2*) encodes a zinc (Zn) transporter that forms a homodimer and transports Zn into intracellular vesicles [4,5,6] in highly specialized secretory cells, where it plays a critical role in regulating vesicle acidification and secretion [6,7]. In the small intestine, ZnT2 is predominantly expressed in Paneth cells in the crypts of Lieberkühn [8]. Interestingly, ZnT2 mRNA has also been documented in the colon [9]; however, the role of ZnT2 in the colon has not been explored. Numerous genetic variants in human ZnT2 that result in loss-of-function (LOF) have been identified [10] which underpins the need to understand the function of ZnT2 in the intestine. In fact, we recently identified two ZnT2 variants, one of which (Exon 7) is associated with enrichment of nosocomial bacteria in the gut and changes in intestinal gene expression consistent with mucosal proliferation and remodeling, while the other (L^293^R) is associated with profound gut dysbiosis, changes in intestinal gene expression consistent with inflammation, and poor growth in preterm infants [11]. Collectively, this suggests ZnT2 function in the host intestinal epithelium plays an important role in maintaining mucosal homeostasis and is critical for robust response to infectious colitis. 

Here, we hypothesized ZnT2 function is a critical determinant of the colonic response to an infectious challenge. We demonstrated ZnT2 is expressed in colonocytes and transports Zn into acidic vesicles. Intriguingly, ZnT2 deletion in mice led to loss of Toll-like receptor 4 (TLR4) expression in colonocytes. Moreover, infection with *Citrobacter rodentium,* a mouse-adapted A/E bacterium used as a model of infectious colitis, illustrated ZnT2 deletion blunted the colonic inflammatory response and was associated with persistent colitis. In addition, ZnT2 attenuation in human colonocytes (HT29 cells) reduced vesicular Zn sequestration and LPS-stimulated lysosome biogenesis, NF-κβ-mediated signaling, expression of proinflammatory cytokines, and inflammation-associated autophagy. Collectively, our data identified novel functions for ZnT2 in colonocytes in modulating mucosal inflammation and support a role for ZnT2 in the ability to appropriately respond to infectious colitis.

## 2. Results

### 2.1. ZnT2 Is Expressed in the Colon and Localized to Acidic Vesicles in Colonocytes

**Figure 1:**
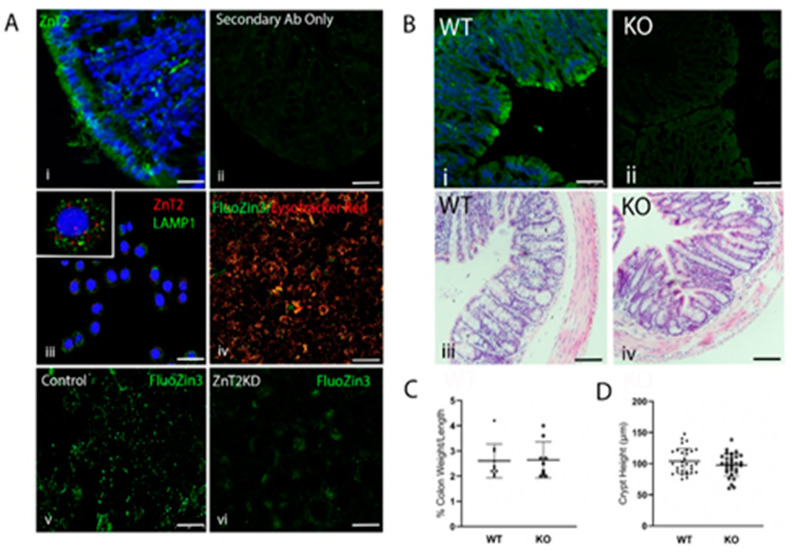
ZnT2 is expressed in acidic vesicles in colonocytes. (**A**) Representative images of (i) ZnT2 (green) and DAPI (blue) in the colon of mice; negative control was incubated with the secondary antibody only (ii). Magnification ×40; scale bars = 50 µm. Representative image of ZnT2 (red) and lysosome associated membrane protein 1 (LAMP1; green, iii) in human colonocytes (HT29 cells). Magnification ×40 (insert, ×63); scale bar =25 µm. Representative image of Lysotracker Red (red) and FluoZin3 (green, iv) in HT29 cells. Magnification ×20; scale bar = 25 µm. Representative images of FluoZin3 (green) in ZnT2-expressing (v) and ZnT2-attenuated (ZnT2KD; vi) HT29 cells. Magnification ×20; scale bar = 25 µm. (**B**) Representative images of ZnT2 (green) and DAPI (blue) in the colon of wild-type (WT; i) and ZnT2-null (KO; ii) mice. Magnification ×20; scale bars = 200 µm. Representative images of the colons stained with H&E of wild-type (WT; iii) and ZnT2-null (KO; iv) mice. Magnification ×20; scale bars = 200 µm. (**C**) Percent colon weight/length in WT and KO mice. Values are means ± SD; *n* = 9–10 mice/genotype. (**D**) Crypt height (µm) in WT and KO mice. Values are means ± SD; *n* = 10 crypts/mouse from 3 mice/genotype.

Immunofluorescent microscopy was used to detect ZnT2 in the colon, which demonstrated a distinct punctate staining pattern most abundant in the apical region of colonocytes (Figure 1A(i,ii)). We next utilized confocal imaging in HT29 cells to refine the sub-cellular localization of ZnT2 in colonocytes. We determined ZnT2 was partially co-localized with lysosome-associated protein 1 (LAMP1; Figure 1A(iii), Pearson’s Coefficient = 0.6), and confirmed the Zn-responsive fluorophore FluoZin3 was predominantly co-localized with the acidic vesicle marker Lysotracker Red (Figure 1A(iv)). Importantly, ZnT2 attenuation using siRNA transfection resulted in depletion of vesicularized Zn pools and subsequent accumulation of labile Zn in the cytoplasm (Figure 1A(v,vi)). This is consistent with observations of ZnT2 localization in other tissues [6,7,12] and supports a role for ZnT2 in Zn transport from the cytoplasm of colonocytes for accumulation into acidic vesicles.

### 2.2. Loss of ZnT2 Does Not Cause Colonic Inflammation or Pathology under Baseline Conditions

To determine the physiological relevance of ZnT2-mediated Zn transport on colon health, ZnT2-null mice (ZnT2KO) were generated and compared to their wild-type (WT) littermates. Loss of ZnT2 in the colon was confirmed (Figure 1B(i,ii)), and under baseline conditions (Figure 1B(iii,iv)), no significant differences in classic hallmarks of colonic inflammation including % colon weight/length (Figure 1C) or crypt height (Figure 1D) were detected in ZnT2KO mice.

### 2.3. Loss of ZnT2 Impairs Response to Infectious Colitis

**Figure 2:**
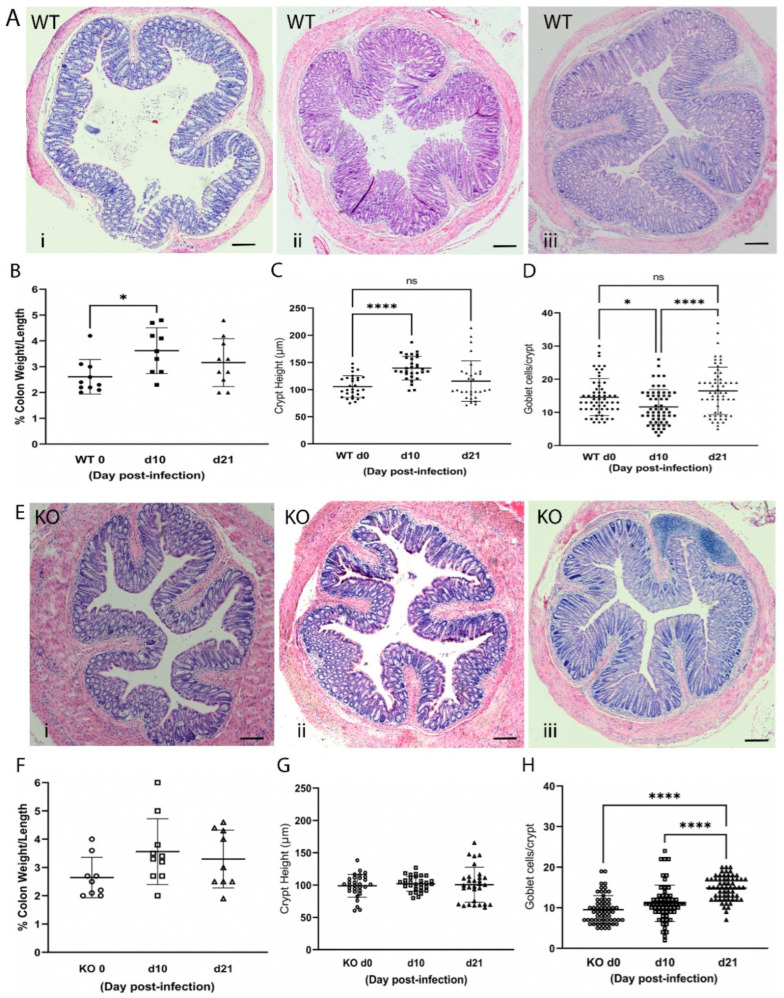
Infection with *Citrobacter rodentium* caused infectious colitis in wild-type (WT) but not ZnT2-null (KO) mice. (**A**) Representative hematoxylin-eosin-stained images of colons from WT mice pre-infection (i), and 10 (ii) and 21 (iii) days post-infection. Magnification ×10; Scale bars = 1 mm. (**B**) Percent colon weight/length in WT mice. Values are means ± SD; *n* = 9–10 mice/genotype; * *p* < 0.05. (**C**) Crypt height (µm) in WT mice. Values are means ± SD; *n* = 10 crypts/mouse from 3 mice/genotype; **** *p* < 0.0001. (**D**) Goblet cell number/crypt in WT mice. Values are means ± SD; *n* = 20 crypts/mouse from 3 mice/genotype; * *p* < 0.05, **** *p* < 0.0001. (**E**) Representative hematoxylin-eosin-stained images of colons from KO mice pre-infection (i), and 10 (ii) and 21 (iii) days post-infection. Magnification ×10; Scale bars = 1 mm. (**F**) Percent colon weight/length in KO mice. Values are means ± SD; *n* = 9–10 mice/genotype. (**G**) Crypt height (µm) in KO mice. Values are means ± SD; *n* = 10 crypts/mouse from 3 mice/genotype. (**H**) Goblet cell number/crypt in KO mice. Values are means ± SD; *n* = 20 crypts/mouse from 3 mice/genotype **** *p* < 0.0001. ns: not significant.

Next, WT and ZnT2KO mice were gavaged with *Citrobacter rodentium* (2 × 10^9^ cfu/mouse) and followed for 21 days. In this model, peak infection occurs by day 10, while resolution should be underway by day 21 [13]. Results from a pilot study documented enrichment of *Citrobacter rodentium* by day 6 post-infection in our model (Supplementary Materials). Histology was used to document effects on the colon in WT and ZnT2KO mice (Figure 2). Our data clearly illustrated a robust response to infection in WT mice. Areas of inflammation and effacement of the colonic epithelium were detected in the WT mice (Figure 2A). At day 10 post-infection, the % colon weight/length (Figure 2B) and crypt height (Figure 2C) were significantly greater (*p* < 0.05 and *p* < 0.01, respectively), while the number of goblets cells/crypt were significantly lower (Figure 2D; *p* < 0.01), illustrating classic hallmarks of colonic hyperplasia and goblet cell depletion associated with infectious colitis. At day 21 post-infection, recovery was well underway as the % colon weight/length was no longer significantly different from that observed pre-infection, and crypt height and goblet cell number were returning to baseline.

**Figure 3:**
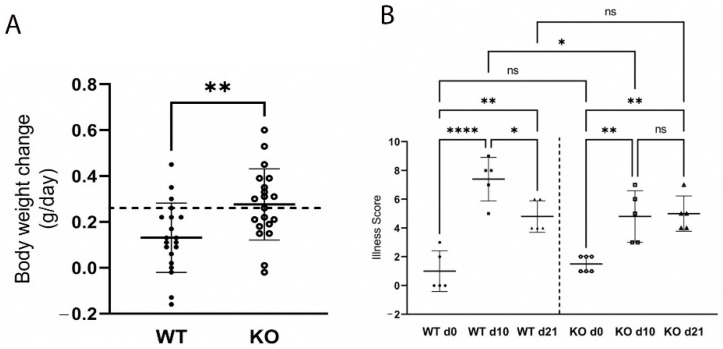
Wild-type (WT) mice gained less weight (**A**) and were more ill (**B**) than ZnT2-null (KO) mice during infectious colitis. A. Values are mean body weight change (g/day) over the first 10 days of infection ± SD; *n* = 19–20 mice/genotype; ** *p* < 0.01. Dashed line represents the mean weight gain (g/day) of non-infected WT and KO mice. B. Illness score in WT and KO mice pre-infection (d0), and 10 (d10) and 21 (d21) post-infection. Values are means ± SD; *n* = 5–6 mice/genotype; * *p* < 0.05, ** *p* < 0.01. **** *p* < 0.0001. ns: not significant.

Moreover, WT mice gained less weight over the course of infection (Figure 3A) which coincided with colonic pathology and significant illness (Figure 3B; *p* < 0.0001), as scored by four key determinants: crypt hyperplasia, loss of colonic goblet cells, surface epithelial injury, and neutrophil infiltration. 

**Figure 4:**
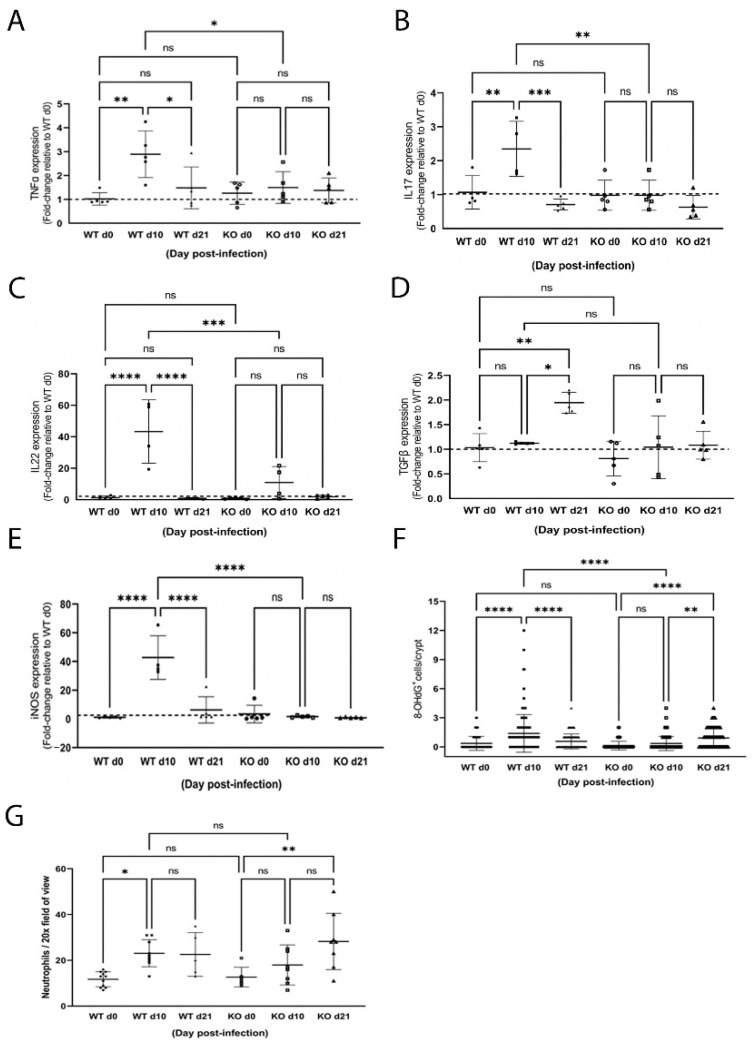
Infection with *Citrobacter rodentium* caused inflammation and oxidative stress in wild-type (WT) but not ZnT2-null (KO) mice. Data represent mean fold-change in TNFα (**A**), IL17 (**B**), IL22 (**C**) and TGFβ (**D**) and iNOS (**E**) mRNA levels normalized to b-actin relative to WT mice pre-infection ± SD in WT and KO mice pre-infection (d0), and 10 (d10) and 21 (d21) post-infection; *n* = 5 mice/genotype; * *p* < 0.05, ** *p* < 0.01, *** *p* < 0.001, **** *p* < 0.0001. (**F**) Number of 8OHdG^+^ cells/crypt ± SD in WT and KO mice pre-infection (d0), and 10 (d10) and 21 (d21) post-infection; *n* = 30 crypts from 3 mice/genotype; ** *p* < 0.01, **** *p* < 0.0001. (**G**) Number of neutrophils/20× field of view ± SD in WT and KO mice pre-infection (d0), and 10 (d10) and 21 (d21) post-infection; *n* = 2–3 fields of view from 3 mice/genotype; * *p* < 0.05, ** *p* < 0.01. ns: not significant.

Associated with illness and histological changes in the colon, WT mice mounted a proinflammatory response (Figure 4) as illustrated by a significant increase in TNFα (Figure 4A; *p* < 0.01), IL17 (Figure 4B; *p* < 0.01) and IL22 (Figure 4C; *p* < 0.0001) expression. Additionally, hallmarks of oxidative stress were detected including a 40-fold increase in iNOS expression (Figure 4E; *p* < 0.0001) that coincided with a significant increase in the number of 8OHdG^+^ cells (Figure 4F; *p* < 0.0001) and infiltrating neutrophils (Figure 4G; *p* < 0.05) 10 days post-infection. All inflammation and oxidative stress indices were resolved by day 21, except for the number of colonic neutrophils, which remained elevated. Moreover, expression of TGFβ was significantly increased at day 21 (Figure 4D; *p* < 0.05), consistent with initiation of a cellular response to resolve colonic inflammation.

In contrast, significantly less colonic inflammation and effacement were detected in ZnT2KO mice, while no significant effect on colonic hyperplasia or goblet cell depletion occurred (Figure 2E–H). Consistent with limited histological changes in the colons, ZnT2KO mice gained more weight than the WT mice over the course of infection (Figure 3A) and were significantly less ill than WT mice (Figure 3B; *p* < 0.05) at peak infection. Additionally, no significant increase in TNFα (Figure 4A), IL17 (Figure 4B), IL22 (Figure 4C) or iNOS (Figure 4E) expression was observed, and neither the number of 8OHdG^+^ cells (Figure 4F) nor infiltrating neutrophils (Figure 4G) were significantly elevated at day 10, indicating the lack of robust pro-inflammatory response. However, there was a significant increase in the number of 8OHdG^+^ cells (Figure 4F; *p* < 0.0001) and infiltrating neutrophils (Figure 4G; *p* < 0.01) at day 21, suggesting a delayed inflammatory response. Moreover, TGFβ expression (Figure 4D) was unaffected, suggesting limited initiation of resolution had occurred by day 21. Additionally, at day 21 the colons of ZnT2KO mice had significantly more TUNEL^+^ cells (24 cells ± 5) compared to WT mice (4 cells ± 2, *p* < 0.05), indicating loss of ZnT2 function was associated with cell death and persistent pathological consequences in the colon. This provides clear evidence that ZnT2 is critical to the ability to mount and resolve a robust pro-inflammatory response to intestinal infection.

### 2.4. ZnT2 Is Important to Initiation of the Proinflammatory Response

**Figure 5:**
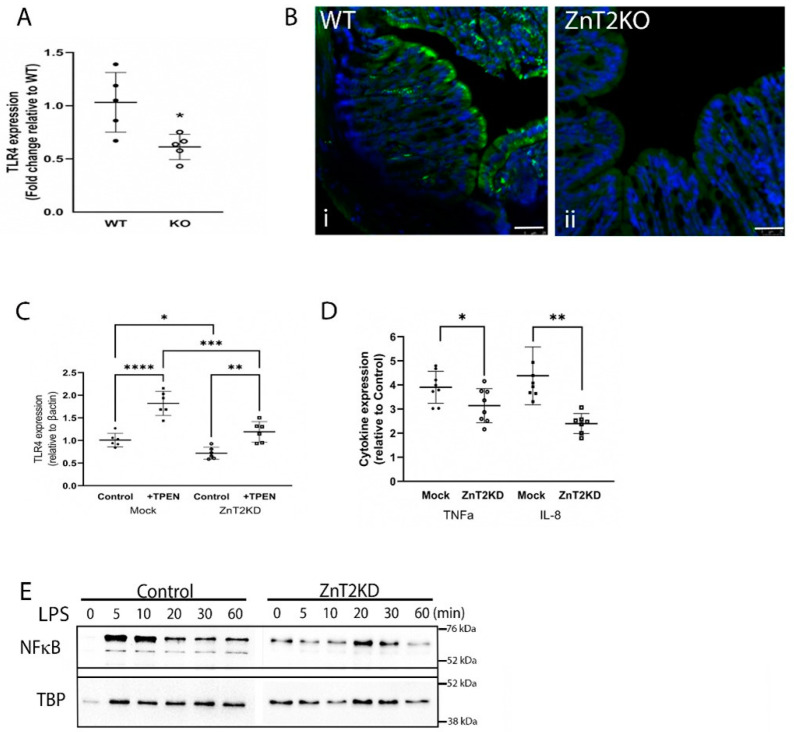
ZnT2 is vital for expression of Toll-like receptor 4 (TLR4) and pathogen-stimulated expression of TNFα and IL8 in the colon. (**A**) Data represent mean fold-change in TLR4 mRNA levels normalized to β-actin relative to WT mice ± SD; *n* = 5 mice/genotype; * *p* < 0.05. (**B**) Representative images of TLR4 expression (green) and DAPI (blue) in the colon of WT (i) and KO (ii) mice. Magnification ×20; scale bars = 50 µm. (**C**) Data represent mean fold-change in TLR4 mRNA levels normalized to β-actin relative to Mock transfected HT29 cells ± SD in ZnT2-expressing (Mock) and ZnT2-attenuated (ZnT2KD) HT29 cells under control conditions or treated with TPEN; *n* = 6 samples/genotype, experiment was repeated twice; * *p* < 0.05, ** *p* < 0.01, *** *p* < 0.001, **** *p* < 0.0001. (**D**) ZnT2-expressing (Mock) and ZnT2-attenuated (ZnT2KD) HT29 cells were treated with LPS (1 µg/mL for 6 h) or left untreated. Data represent mean fold-change in TNFα and IL-8 mRNA levels normalized to β-actin relative to Mock transfected untreated (control) HT29 cells ± SD; *n* = 6 samples/genotype, experiment was repeated twice; * *p* < 0.05, ** *p* < 0.01. (**E**) Representative immunoblot of NF-κβ in the nucleus of Mock-transfected (Control) or ZnT2-attenuated (ZnT2KD) HT29 cells in response to LPS stimulation over 60 min. Membranes were stripped and reprobed for TBP as a normalization control.

Given our findings, we probed consequences of ZnT2 deletion on the colon specifically. We reasoned the lack of robust response to enteric infection suggested loss of ZnT2 impacted either initiation or resolution of colonocyte inflammation. To test the hypothesis that lack of ZnT2 interfered with initiation, we assessed the impact of ZnT2 deletion on TLR4 expression in colonocytes as it is the primary receptor responsible for initiating the inflammatory response to *Citrobacter rodentium*. Surprisingly, TLR4 expression in the colons of ZnT2KO mice was ~50% of that expressed in WT mice (Figure 5A; *p* < 0.05), consistent with extensive staining of TLR4 on the apical membrane of colonocytes in WT mice and very little TLR4 detected in colonocytes of ZnT2KO mice (Figure 5B(i,ii). To determine if this was a direct consequence of lack of ZnT2 in the colonocyte specifically, we attenuated ZnT2 expression in HT29 cells, and consistent with results observed in ZnT2KO mice, TLR4 expression was significantly lower than observed in Mock-transfected cells (Figure 5C; *p* < 0.05). Moreover, a direct role of Zn in the molecular regulation of TLR4 expression was confirmed, as modest Zn chelation using TPEN (10 µM for 30 min) increased TLR4 expression, suggesting cytoplasmic Zn accumulation resulting from ZnT2 attenuation was responsible for loss of TLR4 expression. 

Next, to confirm loss of ZnT2 function in colonocytes impaired activation of the inflammatory response, ZnT2 was attenuated in HT29 cells, and the cells were stimulated with LPS (1 µg/mL for 6h), then expression of TNFα and IL8 as two representative pro-inflammatory cytokines was measured. As shown in Figure 5D, LPS stimulated TNFα and IL8 expression ~4-fold in ZnT2-expressing cells. In contrast, ZnT2-attenuation significantly reduced LPS-mediated stimulation of TNFα (*p* < 0.05) and IL8 (*p* < 0.01) mRNA levels. However, it is noteworthy that ZnT2 attenuation did not eliminate LPS-induced inflammation entirely, which may have been a consequence of residual ZnT2 and/or TLR4 expression in our HT29 model, or the presence of additional regulatory mechanisms. Furthermore, impaired activation of LPS-induced inflammation was associated with delayed and tempered NFκB translocation into the nucleus in ZnT2-attenuated cells (Figure 5E). Collectively this provides evidence that ZnT2-mediated Zn sequestration into lysosomes in colonocytes is critical to buffering cellular Zn pools and is vital for TLR4 expression and robust activation of the mucosal inflammatory response.

### 2.5. ZnT2 Is Important to Activation of Lysosome Biogenesis and Recovery

**Figure 6:**
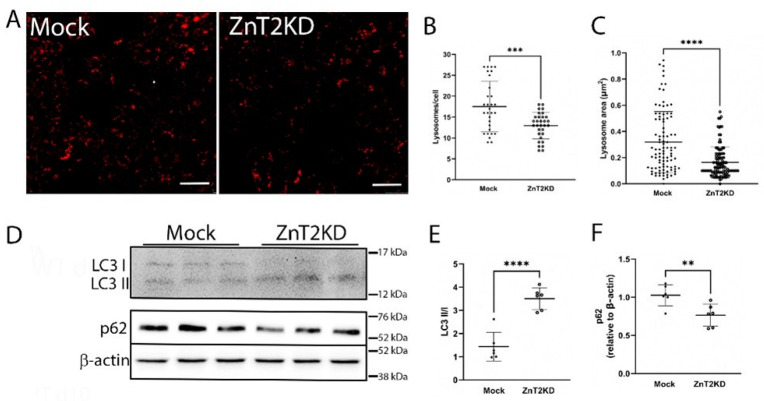
Loss of ZnT2 is associated with fewer and smaller lysosomes but does not affect basal autophagy in HT29 cells. (**A**) Representative image of Lysotracker Red (red) in ZnT2-expressing (Mock) and ZnT2-attenuated (ZnT2KD) HT29 cells. Magnification ×20; scale bar = 25 µm. (**B**) Data represent mean number of lysosomes/cell ± SD; *n* = 25 cells/genotype, experiment was repeated twice; *** *p* < 0.001. (**C**) Data represent mean lysosome area (µm^2^) ± SD; *n* = 100 lysosomes/genotype, experiment was repeated three times; **** *p* < 0.0001. (**D**) Representative immunoblot of LC3 and p62 in ZnT2-expressing (Mock) or ZnT2-attenuated (ZnT2KD) HT29 cells. Membranes immunoblotted for p62 were stripped and reprobed for β-actin as a normalization control. (**E**) Data represent mean ratio of LC3II/LC3I ± SD; *n* = 6 samples/genotype, experiment was repeated twice; **** *p* < 0.0001. (**F**) Data represent mean p62 abundance normalized to β-actin ± SD; *n* = 6 samples/genotype, experiment was repeated twice; ** *p* < 0.01.

Lysosomes play a key role in the ability to both mount and resolve in inflammatory response [14]. Since ZnT2 transports Zn into lysosomes in colonocytes (Figure 1) and ZnT2 is critical for lysosome biogenesis in other cell-types [6,12], we hypothesized ZnT2 is critical for inflammation-induced lysosome biogenesis in colonocytes. Confocal imaging using Lysotracker Red in HT29 cells (Figure 6A) confirmed lysosome number and area were significantly lower (Figure 6B; *p* < 0.001) and smaller (Figure 6C; *p* < 0.0001), respectively, in ZnT2-attenuated HT29 cells compared with Mock-transfected cells. We postulated that fewer and smaller lysosomes would lead to reduced autophagy, and thus we determined effects of ZnT2 attenuation on the ratio of LC3 II/I and p62 abundance as autophagy markers. Surprisingly, we found the ratio of LC3 II/I was significantly elevated, while p62 was significantly reduced, suggesting ZnT2 was not necessary for autophagy under baseline conditions (Figure 6D–F).

**Figure 7:**
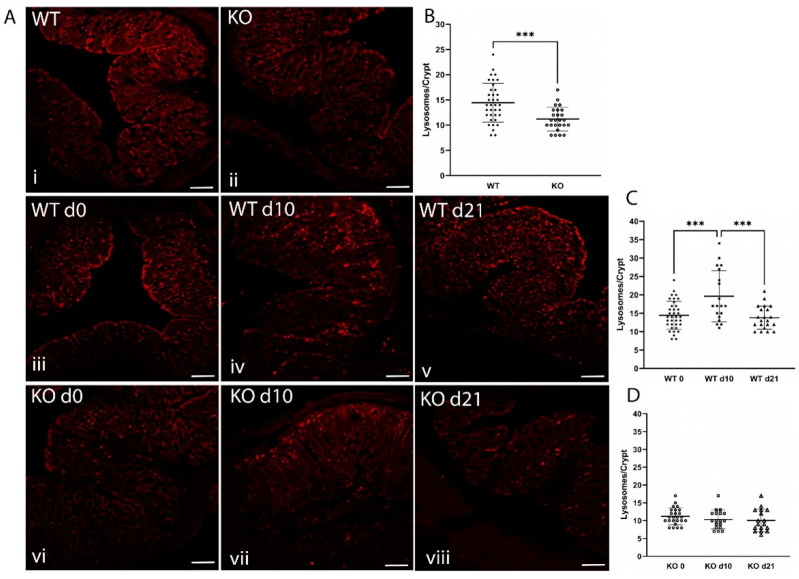
ZnT2 is vital for lysosome biogenesis and the activation of autophagy in colonocytes during infectious colitis in mice. (**A**) Representative images of LAMP1 (red) in the colon of WT (i) and ZnT2-null (KO; ii) mice. Magnification ×20; scale bars = 50 µm. Representative images of LAMP1 (red) in the colons from WT mice pre-infection (iii), and 10 (iv) and 21 (v) days post-infection and KO mice pre-infection (vi), and 10 (vii) and 21 (viii) days post-infection. Magnification ×4; Scale bars = 1 mm. (**B**) Data represent mean number of lysosomes/crypt ± SD; *n* = 25–36 crypts from 3 mice/genotype; *** *p* < 0.001. (**C**) Data represent mean number of lysosomes/crypt in WT mice pre-infection (d0), and 10 (d10) and 21 (d21) post-infection ± SD; *n* = 20–36 crypts from 3 mice/genotype; *** *p* < 0.001. (**D**) Data represent mean number of lysosomes/crypt in KO mice pre-infection (d0), and 10 (d10) and 21 (d21) post-infection ± SD; *n* = 18–25 crypts from 3 mice/genotype.

**Figure 8:**
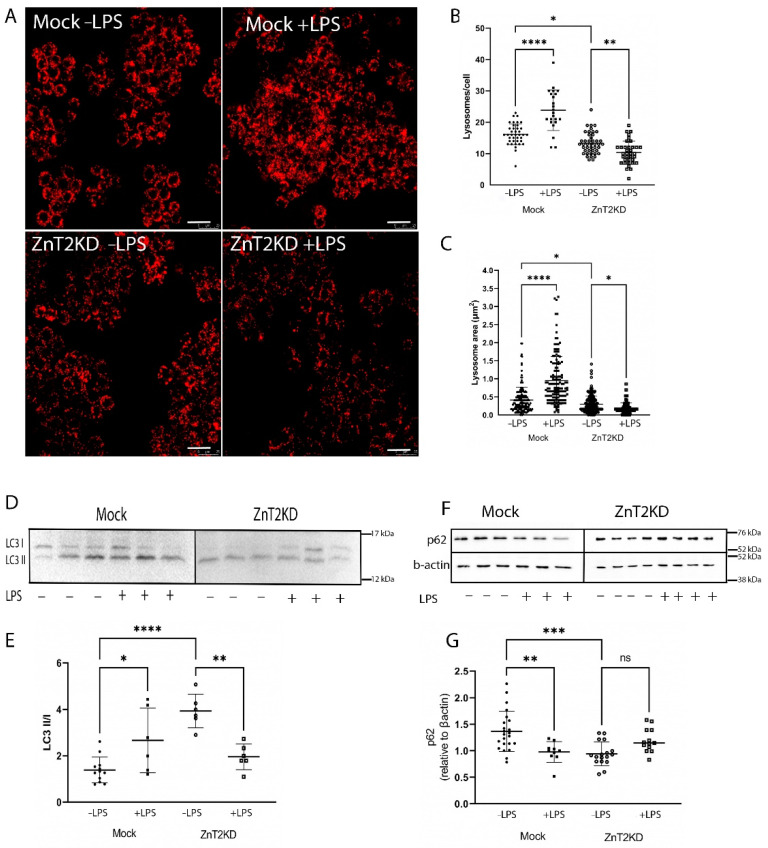
ZnT2 is vital for lysosome biogenesis and the activation of autophagy in HT29 cells. (**A**) Representative images of Lysotracker Red (red) in ZnT2-expressing (Mock) and ZnT2-attenuated (ZnT2KD) HT29 cells treated with LPS (+LPS; 1 µg/mL for 6 h) or left untreated (−LPS). Magnification ×20; scale bar = 25 µm. (**B**) Data represent mean number of lysosomes/cell ± SD in Mock or ZnT2KD cells treated with LPS or left untreated; *n* = 20cells/genotype, experiment was repeated four times; * *p* < 0.05, ** *p* < 0.01, **** *p* < 0.0001. (**C**) Data represent mean lysosome area (µm^2^) ± SD in Mock or ZnT2KD cells treated with LPS or left untreated; *n* = 100 lysosomes/group, experiment was repeated four times; * *p* < 0.05, **** *p* < 0.0001. (**D**) Representative immunoblot of LC3 in ZnT2-expressing (Mock) or ZnT2-attenuated (ZnT2KD) HT29 cells treated with LPS (+) or left untreated (−). Membranes immunoblotted for p62 were stripped and reprobed for β-actin as a normalization control. (**E**) Data represent mean ratio of LC3II/LC3I ± SD; *n* = 6 samples/genotype, experiment was repeated twice; * *p* < 0.05, ** *p* < 0.01, **** *p* < 0.0001. (**F**) Representative immunoblot of p62 in ZnT2-expressing (Mock) or ZnT2-attenuated (ZnT2KD) HT29 cells treated with LPS (+) or left untreated (−). Membranes were stripped and reprobed for β-actin as a normalization control. (**G**) Data represent mean p62 abundance normalized to β-actin ± SD; *n* = 12–18 samples/genotype, experiment was repeated four times; ** *p* < 0.01, *** *p* < 0.001. ns: not significant.

To explore consequences in vivo, we used immunofluorescent imaging of LAMP1 to detect lysosomes in colon sections of WT and ZnT2KO mice. We identified numerous lysosomes in the colonocytes of WT mice (Figure 7A(i)), which were particularly enriched proximal to the apical membrane, while colonocytes in ZnT2KO mice had significantly fewer lysosomes/crypt under baseline conditions (Figure 7A(ii),B; *p* < 0.001). Moreover, lysosome biogenesis was significantly increased in response to infection in the colon of WT mice, and the number of lysosomes returned to baseline by day 21 (Figure 7A(iii–v),C; *p* < 0.001). Consistent with playing a role in an inflammatory response, LPS stimulation significantly increased lysosome number and size (Figure 8A–C; *p* < 0.0001) in Mock-transfected HT29 cells. As expected, inflammation-induced lysosome biogenesis coincided with increased autophagy, as the ratio of LC3 II/I was significantly elevated (Figure 8D,E; *p* < 0.05) and p62 levels were significantly reduced (Figure 8F,G; *p* < 0.001). 

In contrast, ZnT2KO mice were unable to activate colonocyte lysosome biogenesis in response to infection (Figure 7A(vi–viii),D), while ZnT2-attenuation in HT29 cells prevented LPS-stimulated lysosome biogenesis, and lysosome number and size were significantly decreased (Figure 8A–C; *p* < 0.01 and *p* < 0.05, respectively) compared to unstimulated cells. In addition, the ratio of LC3 II/I was significantly reduced (Figure 8D,E; *p* < 0.01) while p62 remained unaffected in response to LPS stimulation in ZnT2-attenuated cells (Figure 8F,G). This provides compelling evidence that while ZnT2 is not required for a basal level of autophagy in colonocytes, ZnT2-mediated vesicular Zn accumulation is critical for lysosome biogenesis and pathogen-stimulated autophagy required to appropriately respond to an infection.

## 3. Discussion

ZnT2-mediated Zn transport into intracellular vesicles plays a critical role in controlling cytoplasmic Zn levels and cellular function in highly specialized secretory cells [6,7,8,12]. Herein, we identified ZnT2 expression in non-secretory colonocytes and provide three principal sets of novel observations regarding their functional significance. First, this is the first study to specifically characterize expression, localization, and function of ZnT2 in the colon. Secondly, we showed ZnT2-mediated cytoplasmic Zn buffering affects TLR4 abundance and permits robust NF-κβ nuclear translocation and inflammatory signaling in response to an infectious challenge. Finally, ZnT2-mediated lysosomal Zn accumulation modulates pathogen-stimulated lysosome biogenesis required for inflammation-induced autophagy and clearance of cellular debris. Collectively this provides compelling evidence that ZnT2-mediated Zn transport in colonocytes is critical for managing mucosal homeostasis and may modulate the risk and severity of infectious colitis.

An exciting and novel finding from this study is that TLR4 expression was affected by ZnT2-mediated cytoplasmic Zn buffering. TLR4 is highly expressed in colonocytes and HT29 cells [15] and regulates initial activation of the inflammatory response to pathogenic microbes [16]. A role for Zn in regulating TLR4 has previously been postulated, as a Zn-enriched diet fed to piglets decreases colonic TLR4 expression and reduces NFκβ signaling and autophagy, which reduces colonic inflammation and weaning-associated diarrhea [17]. This may be a direct effect of Zn or perhaps the consequence of reduced hypoxia-inducible factor 1α (HIF1α) expression [18] which regulates TLR4 expression [19,20]. Further studies are required to understand this relationship. TLR4 activation then mediates MyD88/NF-κβ signaling to increase expression of inflammatory cytokines [21] and further potentiate expression of TLR4 [16]. This process is regulated by cytoplasmic Zn buffering [22] in macrophages and monocytes [22,23,24] and studies by Wu and colleagues show excess Zn interferes with this response [24]. Here, we identified ZnT2 as the Zn transporter responsible for cytoplasmic Zn buffering in colonocytes, as loss of ZnT2 delayed LPS-stimulated nuclear translocation of NF-κβ and reduced inflammatory cytokine expression. This provides a novel paradigm in which a key functional role of ZnT2 in colonocytes is to activate and promote a robust response to clear a bacterial infection. 

In addition to intracellular regulation, extracellular events can also play a role in activating and promoting a robust response. Gut microorganisms can regulate both the innate and adaptive immune responses, and microbial competition and enrichment play key roles in the overall pro- and anti-inflammatory balance of the intestine [25]. For example, enrichment of *Bifidobacterium, Lactobaccillus,* and *Roseburia* is associated with creating an anti-inflammatory environment, while *Firmicutes, Clostridium,* and *Streptococcus* are proinflammatory [25]. We previously showed ZnT2 deletion in mice is associated with enrichment of Bacteriodales S24-7 (*Muribaculaceae*) [8] which is negatively correlated with proinflammatory markers [26], thus it is possible that loss of ZnT2 led to the establishment of an “anti-inflammatory” gut microbiome and played a role in attenuating the inflammatory response. Moreover, composition of the gut microbiome specifically affects expression of TLR4 in mouse colon [27], and in humans, *Bacteriodetes* and *Firmicutes* levels are associated with TLR4 gene expression [28]. While our studies clearly support a role for colonocyte ZnT2 in regulating TLR4, we did not evaluate the gut microbiome or characterize changes in *Citrobacter rodentium* clearance throughout the course of infection, thus advanced transcriptomic and metabolomic studies are required to determine the degree to which the gut microbiome is involved in the response to an infectious challenge in this model. Moreover, Cuesta et al. recently showed that TLR4 deletion itself alters the microbiome [29] and reduces mucosal inflammation, illustrating the complex interplay between the gut microbiome and the host epithelia, and further studies are required to understand the role of ZnT2 on host-microbe interactions.

Consistent with the role of ZnT2 in lysosome biogenesis in secretory cell-types [6,12], we observed a similar role for ZnT2-mediated Zn transport in colonocytes. While the role of ZnT2 appears limited under basal conditions, our data suggest ZnT2 modulates pathogen-stimulated lysosome biogenesis, a role for which is becoming increasingly recognized. Several reports indicate TLR4 activation induces autophagy [21,30,31] during which tumor necrosis factor receptor (TNFR)-associated factor 6 (TRAF6) interacts with Beclin 1 to mediate K63-linked ubiquitination. Ubiquitinated TRAF6 then activates the TGFβ-activated protein kinase 1 (TAK1)/TAK-1-binding protein complex, which phosphorylates the inhibitor of κB (IκΒ) kinase (IKK) complex resulting in Iκβ phosphorylation and degradation, promoting the translocation of NF-κβ into nuclei and the further induction of inflammatory cytokine production [32]. Therefore, if lysosome biogenesis is not stimulated, further potentiation of the inflammatory response is compromised. Moreover, once activated the inflammatory response must be attenuated to maintain tissue homeostasis. Lysosomes are involved in the terminal processing of molecules and are responsible for degradation through autophagy and are required for pathogen-stimulated autophagy to eliminate pathogens, toxins, and dead cells, and contribute to subsequent tissue repair [33,34]. In addition, the downregulation of TLR4-mediated signaling is dependent upon TLR4 internalization and subsequent lysosomal degradation via autophagy, thus inability to activate autophagy leads to accumulation of cellular debris and persistent inflammation. A role for ZnT2 in clearance and tissue repair is consistent with observations of increased neutrophils and dead cells in the colon of ZnT2-null mice during the phase of early resolution; however, by day 21 these differences were modest and further mechanistic studies are required to both understand the molecular role of ZnT2 autophagy, as well as its role in successful resolution of infectious colitis. 

A role for ZnT2 in intestinal health in humans has been previously proposed as genetic variants in ZnT2 have been associated with alterations in the gut microbiome and gene expression in the intestinal exfoliome [11]. A compound substitution in the C-terminus (Exon 7) [35] impairs Zn transport and is associated with enrichment of nosocomial bacteria and enrichment in intestinal genes associated with cell proliferation and remodeling [11]. A second ZnT2 variant (L^293^R) also predicted to impair Zn transport is associated with profound gut dysbiosis, changes in intestinal gene expression consistent with inflammation, and poor growth in preterm infants [11]. As these effects could result from ZnT2-mediated Paneth cell [8], mast cell [36], or colonocyte dysfunction, further studies using tissue-specific ZnT2-null mice are required to understand the function relevance of ZnT2 in each cell-type in managing mucosal homeostasis and response to infectious colitis. Moreover, it is interesting that these consequences present differently from observations in ZnT2KO mice, suggesting there are key molecular differences between complete loss of ZnT2 expression in ZnT2KO mice and the expression of ZnT2 mutants in humans. Nevertheless, this study identified ZnT2 is a novel host-associated epithelial factor associated with mucosal inflammation and suggests manipulating the function of ZnT2 may offer new therapeutic strategies to treat specific intestinal infections and mucosal inflammation.

## 4. Materials and Methods

### 4.1. Animals

All animal protocols were approved by the Institutional Animal Care and Use Committee (IACUC) at the University of Massachusetts Lowell. Male and female heterozygous mice ZnT2^+/−^ were mated to generate wild-type (WT) and ZnT2-null (ZnT2KO) mice as previously described [7]. Male ZnT2KO mice and their WT littermates were weaned onto a purified, powdered control diet (AIN93G; MP Biomedicals, Santa Ana, CA, USA) and maintained on a 12 h light/dark cycle under controlled temperature and humidity and were used for experiments at 6–8 weeks of age.

### 4.2. Intestinal Infection Model

The protocol for inducing mucosal inflammation was adapted from Kennedy and Hartland [13]. Briefly, *Citrobacter rodentium* DBS100 (ATCC, Manassas, VA, USA) was reconstituted according to manufacturer’s instructions and stored at −80 °C. *Citrobacter rodentium* was cultured in Luria Bertani (LB; ThermoFisher, Waltham, MA, USA) liquid media overnight at 37°C with shaking at 250 rpm. The following day, the culture was diluted to an OD_600_ of 0.05 in 100 mL of LB and allowed to grow until log phase (OD_600_ 0.4–0.5). Then, 2 × 10^9^ cfu of *Citrobacter rodentium* were centrifuged at 4500× *g* for 5 min to pellet bacteria and bacteria were resuspended in sterile PBS (50 µL). Bacterial inoculums used for gavage were serially diluted on Luria Broth agar plates to confirm the number of cfu administered. 

After 2 weeks on control diet, WT (*n* = 20) and ZnT2KO (*n* = 20) mice were fasted for 8 h and orally gavaged with 2 × 10^9^ cfu *Citrobacter rodentium*. Mice were weighed every other day and monitored for signs of illness or distress and symptoms were scored (see below). Mice were euthanized 10 d post-infection (*n* = 10/genotype) to document effects at peak infection, or 21 d post-infection (*n* = 10/genotype) to document effects during resolution [13]. In addition, a cohort of mice (*n* = 10/genotype) were orally gavaged with 50 µL of sterile PBS for comparison (Control). Mice were euthanized via CO_2_ asphyxiation. Blood was drawn via cardiac puncture into heparinized microfuge tubes and plasma was isolated by centrifugation at 2000× *g* and stored at −80 °C. The entire intestine (small intestine, cecum, colon) was dissected, and the full length was measured. The small intestine and the colon were separated by dissecting either side of the cecum and gently perfused with sterile PBS. Perfusate was removed and the tissue gently blotted, and the weight and length of the small intestine and the colon were measured. The colon was cut into thirds; the proximal third was stored in TRIzol Reagent (ThermoFisher, Waltham, MA, USA) at −80 °C until RNA extraction, the middle third was snap frozen and stored at −80 °C, and the distal third was fixed in phosphate-buffered paraformaldehyde (4%), pH = 7.4 at 4 °C for 18 h for histology. 

### 4.3. Histological Analysis

Fixed distal colon segments were processed then stained with hematoxylin (ThermoFisher, Waltham, MA, USA) and eosin(ThermoFisher, Waltham, MA, USA) (H&E) or Alcian blue (ThermoFisher, Waltham, MA, USA) or used for immunofluorescent imaging, as previously described [8]. Crypt height was measured in H&E-stained sections (*n* = 10 crypts/mouse) visualized at 4× magnification by drawing a bisecting line from the base of the crypt to the lumen using ImageJ (https://imagej.nih.gov/ij/ (accessed on 23 August 2022)) in 3 mice/group, as previously described [8]. Sections were stained with Alcian blue to visualize goblet cells, and the number of goblet cells/crypt (*n* = 20 crypts/mouse) visualized at 10x magnification were counted in 3 mice/group, as previously described [8]. A TACS-XL Basic In Situ Apoptosis (TUNEL) detection kit (Trevigen, Inc., Gaithersburg, MD, USA, 4828-30-BK) was used to identify apoptotic cells. The number of cells stained positive for TUNEL/section at d21 (20× magnification) were counted (*n* = 3 mice/genotype), as previously described [7].

### 4.4. Illness Score

Illness score determination was adapted from Bouladoux and colleagues [37] and Kang and colleagues [38]. The total illness score assessed four key determinants of classical colonic pathology: crypt hyperplasia, loss of goblet cells, surface epithelial injury, and neutrophil infiltration. Crypt hyperplasia was determined from high quality H&E sections as measured above. The number of goblet cells in intact crypts were counted in high-quality Alcian blue stained sections as measured above. Surface epithelial injury was visually scored in high quality H&E sections by two independent investigators as follows: 0 = normal surface epithelium; 1 = occasional areas of epithelial exfoliation; 2 = frequent areas of epithelial exfoliation; 3 = extensive areas of epithelial ulceration. Neutrophils were visualized using immunofluorescent imaging (described below) and the number of neutrophils were counted in 2–3 fields of view/mouse (magnification = 20×, *n* = 3 mice/group).

### 4.5. Cell Culture and Transfection

Human colorectal adenocarcinoma cells (HT29; Manassas, VA, USA, HTB-38™) were used as a human colonocyte model and were maintained in DMEM supplemented with 10% fetal bovine serum, 0.2% sodium bicarbonate and 1% penicillin-streptomycin at 37 °C under 5% CO_2_. Cells were treated with 1µg/mL LPS prepared in serum-free media for up to 16 h where indicated. *siRNA-mediated ZnT2 attenuation*- Cells were seeded in 6-well plates at a density of 2 × 10^5^ cells/mL in antibiotic-free medium and grown for at least 24 h. When ~40% confluent, cells were transfected with 100 pmol of ZnT2-specific siRNA (5′ GGUCAUACACGGGAUCUCUtt-3′; 5′-AGAGAUCCCGUGUAUGACCgg-3′; (ThermoFisher, Waltham, MA, USA) using Lipofectamine 2000 (ThermoFisher, Waltham, MA, USA) at an oligonucleotide/transfection reagent ratio of 20:1 in Opti-MEM (ThermoFisher, Waltham, MA, USA) for 5 h, after which Opti-MEM was replaced with antibiotic-free media. Cells exposed to Lipofectamine 2000 were used as controls. Successful KD was confirmed by qPCR and immunoblotting as described below. 

### 4.6. Immunofluorescent Imaging

#### 4.6.1. Tissues

The following primary antibodies were used for immunofluorescent imaging: anti-ZnT2 (1 µg/mL) [39], anti-LAMP1 (2 µg/mL; Abcam, Waltham. MA, USA, ab25630), anti-Toll-like receptor 4 (TLR4, 2 µg/mL; Abcam, Burlingame, CA, USA, ab13556), anti-neutrophil antibody (1:100; Abcam, Burlingame, CA, USA, ab2557), and anti-8OHdG (5 µg/mL; Abcam, Burlingame, CA, USA, ab48508). Secondary antibodies used were Alexa Fluor^®^ 488- or Alexa Fluor^®^ 568-conjugated goat anti-rabbit or anti-mouse IgG (2 µg/mL; ThermoFisher, Carlsbad, CA, USA) and sections were counterstained with 4′,6-diamidino-2-phenylindole (DAPI) nuclear stain (1:10,000; ThermoFisher, Waltham, MA, USA, D1306) for 15 min at room temperature (RT). Coverslips were rinsed in PBS and mounted in ProLong Diamond and sealed with nail polish. Sections were examined using the Leica DM IL LED microscope equipped with a digital Leica DFC425 camera (Leica Microsystems, Buffalo Grove, IL, USA) or the Leica Inverted Confocal Microscope SP8 (Leica Microsystems, Buffalo Grove, IL, USA) and images were saved as .jpeg files. The number of LAMP1^+^ vesicles/crypt and 8OHdG^+^ cells/crypt in 3 mice/group were counted.

#### 4.6.2. Cells

HT29 cells were seeded onto glass coverslips and cultured until ∼60% confluent. Cells were fixed in 4% w/v phosphate-buffered paraformaldehyde (pH 7.4) for 10 min, washed in PBS and permeabilized with 0.2% Triton X-100 (ThermoFisher, Waltham, MA, USA) in PBS for 10 min. Non-specific binding was avoided by incubating with blocking buffer (1% BSA, 5% donkey serum, 0.2% Triton X-100 diluted in PBS) for 20 min, followed by incubating with affinity-purified rabbit anti-ZnT2 antibody (1 µg/mL) for 1 h in blocking buffer. Following three washes with PBS, ZnT2 antibody was detected with Alexa Fluor^®^ 568-conjugated goat anti-rabbit IgG (4 µg/mL; ThermoFisher, Waltham, MA, USA) for 20 min, shielded from light. To determine sub-cellular localization, cells were reblocked and then incubated with mouse anti-LAMP1 (2 µg/mL; Abcam, Burlingame, CA, USA, ab25630) for 1 h at RT, washed three times in PBS, followed by detection with Alexa Fluor^®^ 488-conjugated goat anti-mouse IgG (1 µg/mL; ThermoFisher, Waltham, MA, USA) for 20 min. Finally, nuclei were stained with DAPI (1:10,000; ThermoFisher, Waltham, MA, USA) for 15 min at RT. Coverslips were rinsed in PBS and mounted in ProLong Diamond and sealed with nail polish. Imaging was performed using LEICA SP8 confocal microscope with 63× oil objective (Leica Microsystems, Buffalo Grove, IL, USA). Digital images were captured in frame or stack sequential mode to eliminate potential interferences between fluorophores and exported as .jpeg images. To quantify the subcellular colocalization of ZnT2 with LAMP1, a total of 25 single cell images from two independent experiments were used. Pearson’s correlation coefficients were determined using the LASX software analytical tool (Leica Microsystems, Buffalo Grove, IL, USA). The value ranges between +1 to −1, with +1 illustrating a positive correlation, −1 a negative correlation, and zero illustrating no correlation [40].

#### 4.6.3. Lysosome Number

HT29 cells (ZnT2-attenuated or control) were cultured on glass coverslips in 24 well plates for 48 h. Cells were washed with PBS and treated with Lysotracker Red DND-99 (75 nM; ThermoFisher, Waltham, MA, USA, L7508) in Opti-MEM for 1 h, shielded from light. The fluorescence of Lysotracker Red (emission 577 nm/excitation 590 nm) in live cells was captured using the Leica Inverted Confocal Microscope SP8 (magnification = 63× oil), and number of lysosomes in all cells captured in five random fields of view, from three different samples/group were counted. The experiment was repeated three times.

#### 4.6.4. Intracellular Zn pools

HT29 cells (ZnT2-attenuated or control) were cultured on glass coverslips in 24 well plates for 48 h. Cells were washed with 1X PBS and treated with FluoZin3-AM (ThermoFisher, Waltham, MA, USA, F24195; emission 485 nm/excitation 520 nm) in Opti-MEM containing 0.02% Pluronic F-127 (ThermoFisher, Waltham, MA, USA, P3000MP) for 1 h at 37 °C, as recommended by the manufacturer. Subsequently, cells were rinsed twice with PBS for 5 min each at RT with constant shaking. The fluorescence of FluoZin3-AM in live cells was imaged using the Leica Inverted Confocal Microscope SP8 (magnification = 63× oil) in three random images from three different samples/group. The experiment was repeated three times. As a negative control, HT29 cells were treated with the Zn chelator, N, N, N’, N’ -tetrakis (2-pyridylmethyl)ethylenediamine (TPEN; 10 µM, ThermoFisher, Waltham, MA, USA) for 30 min prior to FluoZin3-AM treatment. 

### 4.7. Gene Expression

RNA was isolated from the colon (*n* = 5 mice/group) or HT29 cells (*n* = 3 samples/group, repeated at least twice) using TRIzol reagent ThermoFisher, Waltham, MA, USA following manufacturer’s instructions. RNA purity was determined using a NanoDrop 2000 (ThermoFisher, Waltham, MA, USA), RNA quality was determined by electrophoresis through a 1% agarose gel, and RNA concentration was determined using the Qubit fluorometer (ThermoFisher, Waltham, MA, USA). Total RNA (1 µg) was reverse transcribed to cDNA using iScript™ Reverse Transcription Supermix (Biorad, Hercules, CA, USA) then utilized for quantitative PCR using ssoAdvanced Universal SYBR Green Supermix (Biorad, Hercules, CA, USA) with the following conditions: 1 cycle at 95 °C for 30 s, and 40 cycles at 95 °C for 15 s, 60 °C for 30 s using a Real Type PCR detection system (MyiQ2, (Biorad, Hercules, CA, USA). Data were analyzed using the real-time PCR analysis software Bio-Rad iQ5 (Biorad, Hercules, CA, USA). Relative gene expression was calculated using the 2^−ΔΔCt^ method, where Ct represents the cycle threshold [41]. Δ^Ct^ values were calculated as the difference between the target gene and β-Actin as a normalization control. The melting curve was evaluated for all primer pairs and primer sequences are listed in Table 1.

### 4.8. Immunoblotting

Colon homogenates, cell homogenates, and crude membrane fractions were generated as previously described [12]. Nuclear fractions were generated using the NE-PER Nuclear and Cytoplasmic Extraction kit (ThermoFisher, Waltham, MA, USA 78833) per manufacturer’s instructions. Protein (20 µg) was diluted in Laemmli sample buffer (Biorad, Hercules, CA, USA) containing 100 mM dithiothreitol (DTT, ThermoFisher, Waltham, MA, USA), electrophoresed, and proteins were detected by immunoblotting as previously described [8]. Primary antibodies used: anti-ZnT2 (1:1000) [39], anti-HA (0.1 µg/mL; ThermoFisher, Waltham, MA, USA 12CA5), anti-LC3B (1:1000; Abcam, Burlingame, CA, USA, ab51520), sequestrin/p62 (1:1000; ThermoFisher, Waltham, MA, USA, MA5-27800), anti-p65 (1:1000, Cell Signaling Technologies, Danvers, MA, USA, 8242). Primary antibodies were visualized with anti-rabbit IgG-horseradish peroxidase (1:10,000; ThermoFisher, Waltham, MA, USA, NA934) or anti-mouse IgG-horseradish peroxidase (1:20,000; ThermoFisher, Waltham, MA, USA, 31430). Where indicated, membranes were stripped and re-probed using rabbit anti-βactin (1:10,000; ThermoFisher, Waltham, MA, USA, MA5-15379) or anti-TATA binding protein (TBP) antibody (1:1000; Cell Signaling Technologies, Danvers, MA, USA 44059) as total protein and nuclear loading controls, respectively. Proteins were detected with SuperSignal West Femto Maximum Sensitivity Substrate (ThermoFisher, Waltham, MA, USA, 34096). Images were captured using ChemiDoc MP (Bio-Rad, Hercules, CA, USA) Imaging System. Band density was measured using Image J software (https://imagej.net accessed on 23 August 2022).

### 4.9. Statistical Analysis

Data are presented as mean ± SD. For HT29 experiments, all samples were run in at least triplicate and all experiments were repeated at least two times. Statistical comparisons were performed using Student’s *t*-test (Kruskal–Wallis for data not normally distributed) or one-way ANOVA (data not normally distributed) where appropriate (Graph Pad Prism; Berkeley, CA, USA) and a significant difference was demonstrated at *p* < 0.05.

## Figures and Tables

**Table 1:** Primer sequences used for qPCR.

Gene	Primer	Sequence (5′ to 3′)
Mouse *TNFA*	Forward Reverse	TTGTCTACTCCCAGGTTCTCT GAGGTTGACTTTCTCCTGGTATG
Mouse *TGFB1*	Forward Reverse	GGTGGTATACTGAGACACCTTG CCCAAGGAAAGGTAGGTGATAG
Mouse *IL6*	Forward Reverse	GATAAGCTGGAGTCACAGAAGG TTGCCGAGTAGATCTCAAAGTG
Mouse *IL17A*	Forward Reverse	CCACGTTTCTCAGCAAACTTAC TGTGGAGGGCAGACAATTC
Mouse *IL22*	Forward Reverse	CGACCAGAACATCCAGAAGAA GAGACATAAACAGCAGGTCCA
Mouse *NOS2*	Forward Reverse	CCGCCGCTCTAATACTTA TTCATCAAGGAATTATACAGGAA
Mouse *TLR4*	Forward Reverse	CCAGGTGAGCTGTAGCATTTA GAGCAAACAGCAGAGGAAGA
Mouse *SLC30A2*	Forward Reverse	GGTTACAGATGCAAGAGGTAAGA TGGCCTGCAATGACAGATATAA
Mouse *ACTB*	Forward Reverse	AGGGAAATCGTGCGTGACAT GAACCGCTCGTTGCCAATAG
Human *TLR4*	Forward Reverse	TCTAAGCCAGTGTCTCCATTTAC GAGGTCTGCCAACAAGGTATTA
Human *CXCL8*	Forward Reverse	CTTGGCAGCCTTCCTGATTT GGGTGGAAAGGTTTGGAGTATG
Human *TNFA*	Forward Reverse	CTCTTCTGCCTGCTGCACTTTG ATGGGCTACAGGCTTGTCACTC
Human *SLC30A2*	Forward Reverse	CCTGCGAGTGGTAGAACATT CTCAGCGGGTATAAAGCTAGTG
Human *ACTB*	Forward Reverse	CACCATTGGCAATGAGCGGTTC AGGTCTTTGCGGATGTCCACGT

## Data Availability

The data presented in this study are openly available in Figshare at 10.6084/m9.figshare.20498913.

## Supplementary Materials

The following supporting information can be downloaded at: https://www.mdpi.com/article/10.3390/ijms231911467/s1.
