# Supplementary material for: ZnT2 Is Critical for TLR4-Mediated Cytokine Expression in Colonocytes and Modulates Mucosal Inflammation in Mice
Source: Int J Mol Sci. 2022 Sep 28;23(19):11467. doi: 10.3390/ijms231911467 (PMC9570081; doi:10.3390/ijms231911467)
Supplement: Supplementary file 1 [file ijms-23-11467-s001.zip › ijms-1907140-supplementary.pdf]

Supplementary Figure S1. Colonization of *Citrobacter rodentium* cultured from fecal extracts of mice.

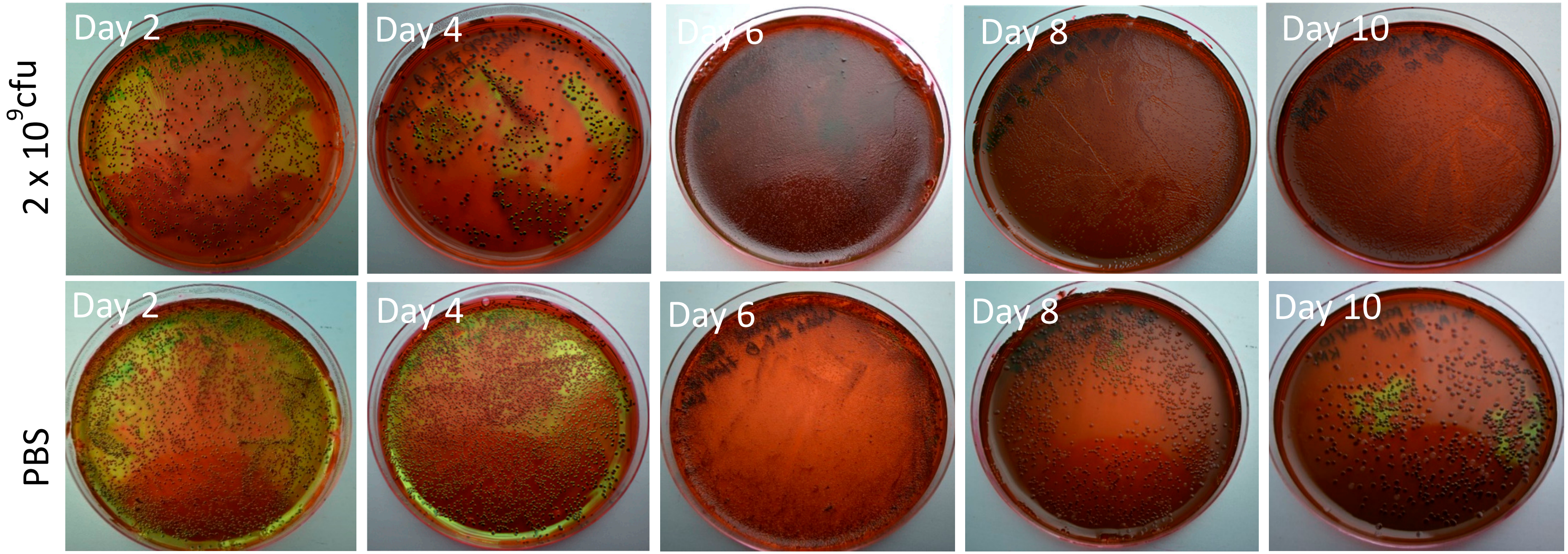


Mice were fasted for 8 hours and gavaged with either *Citrobacter rodentium* or PBS (in 200 uL). Fresh fecal pellets were collected in a sterile microfuge tube and PBS was added to a final concentration of 50 mg/mL. Feces were vortexed to emulsify and serially plated onto EMB agar and cultured for 24 h at 37C. The density of bacterial growth was compared across time to ensure infection (*E.coli* appears metallic green, while *C. rodentium* appears as tiny dusky pink colonies). Note the robust colonization by Day 6 in infected mice.
